# Acid-base status of the blood contained in the cardiotomy reservoir during deep hypothermic circulatory arrest at 18 °C

**DOI:** 10.1051/ject/2025014

**Published:** 2025-09-15

**Authors:** Sylvain Diop, Marwan Nader, Elie Fadel, Maria Cristina Kassab, Hamdi Ghadbane, Iolanda Ion, Jacques Thes

**Affiliations:** 1 Department of Anesthesiology, Marie Lannelongue Hospital, Paris Saint Joseph Hospital 133 Avenue de la Résistance 92350 Le Plessis Robinson France; 2 Cardiothoracic Intensive Care Unit, Marie Lannelongue Hospital, Paris Saint Joseph Hospital 133 Avenue de la Résistance 92350 Le Plessis Robinson France; 3 Department of Vascular and Thoracic Surgery, Marie Lannelongue Hospital, Paris Saint Joseph Hospital 133 Avenue de la Résistance 92350 Le Plessis Robinson France; 4 Perfusionist Team, Department of Anesthesiology, Marie Lannelongue Hospital, Paris Saint Joseph Hospital 133 Avenue de la Résistance 92350 Le Plessis Robinson France

**Keywords:** Deep hypothermic circulatory arrest, Pulmonary artery endarterectomy, Acid base, pH-stat, Alpha-stat, Cerebral blood flow

## Abstract

*Background*: During deep hypothermic circulatory arrest (DHCA) for pulmonary artery endarterectomy (PAE), the blood volume stored in the cardiotomy reservoir circulates through the oxygenator via the arterial shunt line, where it remains oxygenated and decarboxylated. The aim of the study was to investigate the change in the acid-base balance of the blood contained in the cardiotomy reservoir during DHCA. *Methods*: A four-month retrospective analysis was conducted on patients undergoing PAE. The sweep gas inflow and the inspired fraction of O_2_ were kept constant throughout the duration of DHCA. Arterial blood gases were sampled at the beginning and at the end of the DHCA and were analyzed according to the alpha-stat and pH-stat strategies. *Results*: Twenty-four patients were included with a mean age of 59.2 (±15.7) years. The mean duration of DHCA was 15.2 (±4.1) min and the mean sweep gas inflow was 1.4 (±0.8) L/min. Initial pH and PaCO_2_ were 7.31 (±0.09) and 43.2 (±9.9) mmHg, respectively, and final pH and PaCO_2_ were 7.51 (± 0.14), *p* < 0.001 and 23.4 (±11.9) mmHg, *p* < 0.001. There was a significant correlation between the sweep gas inflow and the post-DHCA pH (*r* = 0.797). *Conclusion*: The pH increases significantly during the DHCA according to the sweep gas inflow. Decreasing the sweep gas inflow between 0.5 and 1.0 L/min allows for limiting the pH variation during the DHCA period.

## Introduction

Pulmonary artery endarterectomy (PAE) is a complex surgery performed under cardiopulmonary bypass (CPB) to treat certain phenotypes of chronic thromboembolic pulmonary hypertension [1]. To allow the surgeon to adequately remove the clot material from inside the pulmonary arteries, a bloodless surgical field is required and obtained through one, or more, short periods of deep hypothermic circulatory arrest (DHCA) (CA being the complete interruption of CPB) after having cooled the body to 18 °C [1]. During DHCA, the blood volume, outside the residual blood volume of the body vessels and tissue, is stored in the cardiotomy reservoir. During that period, according to our local CPB management, the blood circulates through the oxygenator via the arterial shunt line, at a flow ranging from 0.4 to 0.8 L/min and remains oxygenated and decarboxylated on account of the sweep gas inflow. Recommendation from the American Society of Extracorporeal Technology advocates setting up the sweep gas flow to maintain blood at normocapnic level [2]. However, to the extent of our knowledge, there is no clinical data regarding the composition of the blood reinjected after this period of DHCA. We hypothesize that the composition of the blood changes dynamically during the time of DHCA. As pH and partial pressure of carbon dioxide (PaCO_2_) dynamically modify cerebral blood flow, it could have some clinical impact on brain perfusion [3–7]. If present, acid-base disturbances occurring during DHCA could be anticipated and eventually corrected before resuming CPB, mainly by lowering the rate of sweep gas inflow. So, this study aimed to investigate the change in the acid-base balance and the electrolyte composition of the blood contained in the cardiotomy reservoir, during circulatory arrest in a patient undergoing PAE.

## Methods

### Patients

A retrospective analysis was performed on all patients undergoing PAE during this period from January to April 2024. During this period, to improve our local CPB management, arterial blood gas was sampled at the beginning and the end of the first DHCA to better adjust the rate of sweep gas flow. Patients under 18 years old, pregnant women, and patients (or their relatives) who refused the use of their medical data for study purposes were not included.

### Ethics

This study has been approved by the ethical committee of the Marie Lannelongue Surgical Center (Ethical Committee IRB N° 00012157), Paris Saint Joseph Hospital Group, Le Plessis Robinson, France. According to French law, all patients included were informed of the use of their medical data for the study purposes, and their right to refuse or withdraw from participation at any time.

### Anaesthetic and cardiopulmonary bypass management

#### Anesthesia management

Anesthesia care was provided according to the local department protocol. Invasive blood pressure monitoring of all patients was achieved by inserting a left femoral arterial catheter under local anesthesia before the induction of general anesthesia (GA). Patients were then put under GA via intravenous injections of the following: Sufentanil at 0.3 μg/kg, Etomidate at 0.3–0.4 mg/kg, and Rocuronium at 0.4 mg/kg. GA was maintained by a target-controlled infusion (TCI) of Propofol and Sufentanil, and a continuous infusion of the neuromuscular blocking agent to avoid shivering during the cooling and rewarming processes. Patients were put under mechanical ventilation with a tidal volume of 6–8 mL per kg of predicted body weight, and a positive end-expiratory pressure (PEEP) of 5 cmH_2_O. The respiratory rate was adjusted to maintain an arterial carbon dioxide pressure (PaCO_2_) ranging from 35 to 45 mmHg, and the fraction of inspired O_2_ (FiO_2_) was adjusted to target a blood oxygen saturation (SpO_2_) above 94%, before and after bypass. A pulmonary arterial catheter (Swan-Ganz COmbo V 7.5 Fr, Edwards Lifesciences, Irvine, CA 92614 USA) was inserted, right after the induction of general anesthesia, under ultrasound guidance, allowing continuous measurements of the pulmonary pressure, the cardiac output, and the venous oxygen saturation, allowing optimal hemodynamic management both before initiation and after separation from CPB. During the procedure, the catheter is pulled back into the right ventricle on surgical demand to not interfere with material removal into the pulmonary arteries. The catheter is then inserted into the right pulmonary artery by the surgeon. A single dose of 120 mg of methylprednisolone was injected, for cerebral protection, during the cooling period, once the patient’s temperature reached 25 °C. A single dose of 1000 mg of sodium thiopental was administered when the temperature reached 20 °C to suppress any residual cerebral activity before the DHCA.

#### CPB management

CPB was established with a nonpulsatile centrifugal pump (Essenz^TM^ Perfusion System, Livanova^TM^, Eastbourne Terrace, London, England W2 6LG) and blood cardioplegia. The circuit consisted of a membrane oxygenator (8F, Inspire membrane oxygenator, Livanova^TM^, Eastbourne Terrace, London, England W2 6LG), a pump, and cannulas. CPB prime consisted of 500 mL of albumin at 4%, 1000 mL of Ringer’s lactate solution, and 250 mL of a sodium bicarbonate solution at 1.4%. The venous cannulas were inserted in each vena cava, and the arterial cannula was inserted into the ascending aorta. Before cooling, an asanguineous priming of CPB is done to obtain hemodilution, with a hemoglobin level target between 9 and 10 g/dL and, if needed, by total blood sequestration (the volume sequestrated would have to be replaced with an equivalent volume of crystalloid). At the beginning of CPB, the membrane fraction of O_2_ (F_m_O_2_) was set to 60%, and was then increased to 80% or above during the cooling process (as soon as the esophageal temperature reached 35 °C). The patient’s core temperature was monitored at two sites: the bladder and the esophagus. Patients were only cooled by means of the oxygenator heat exchanger (without exceeding a gradient of 10 °C between the arterial outlet temperature and the venous inflow temperature) at a rate of approximately 1 °C every 2–3 min. On average, it takes between 40 and 60 min to obtain the targeted temperature. Once the 30 °C threshold was reached, the cardiac index (CI) was progressively lowered at an average rate of 0.05–0.1 L/min/m^2^ per degree Celsius to reach 1.8 L/min/m^2^ at 18 °C [8]. The temperature was then maintained at 18 °C for the duration of the DHCA. Hyperkalemic blood cardioplegia was administered through the aortic root for myocardial protection immediately after aortic cross-clamping and was repeated every 20 min. Between each circulatory arrest, a reperfusion period of at least 10 min was respected. The CI was then progressively increased during the rewarming phase, at the same rate, once the 20 °C threshold was reached, to restore the patient’s baseline CI. Rewarming was achieved at a rate inferior to 0.5 °C per minute, without exceeding a gradient of 10 °C between the arterial outlet temperature and the venous inflow temperature, when the arterial temperature was below 30 °C, and without exceeding a gradient of 5 °C when the temperature was above 30 °C [9]. Mean arterial pressure was maintained between 60 and 80 mmHg with the help of a continuous infusion of norepinephrine (0.2 mg/mL), if necessary. Acid-base management was achieved through an alpha-stat strategy during both cooling and rewarming.

#### Management of the CPB during circulatory arrest

At the time of the circulatory arrest, the revolutions per minute (RPM) of the spinning pump was decreased to 1500 RPM. The arterial cannula was then clamped. The blood was passively drained into the cardiotomy reservoir via the venous lines. The blood continued to circulate through the oxygenators via the arterial shunt line, at a flow ranging from 0.4 to 0.8 L/min. The sweep gas inflow was determined by the perfusionist in charge during cooling. It was kept constant throughout the first DHCA. The F_m_O_2_ ranged from 80% to 100% according to our local protocol. At the end of circulatory arrest, the arterial cannula was unclamped, and the pump RPM progressively increased to its initial level to restore a cardiac index of at least 1.8 L/min/m^2^.

#### Blood samples during circulatory arrest

The first blood gas was sampled on the arterial shunt line at 18 °C just before the beginning of the DHCA. A second one was sampled on the same site at the end of the DHCA, just before resuming CPB. The blood volume in the cardiotomy container, the sweep gas inflow, and the F_m_O_2_ were systematically recorded. Measurements were done only on the first circulatory arrest. All blood gases were immediately analyzed according to the alpha stat and the pH-stat strategies, with the ABL90 FLEX PLUS^©^ Radiometer (Copenhagen, Denmark) as the blood gas analyzer.

### Data collection

The following data were recorded in an anonymized file: demographic data, medical history, and preoperative cardiac evaluation. The duration of CPB, aortic clamping, and circulatory arrest times were recorded. The volume of blood contained in the cardiotomy reservoir, the sweep gas inflow, the arterial shunt line flow, and the F_m_O_2_, were recorded during the period of DHCA. The values of arterial blood gas, corrected and uncorrected to the temperature, performed at the beginning and the end of the DHCA were recorded. The strong ion difference (SID) was also reported and calculated as follows:

$$\mathrm{SID}\;={(\mathrm{Na}}^{+}+K^{+})-{\mathrm{Cl}}^{-}.$$

### Statistical analysis

Qualitative data are expressed as numbers and percentages, and quantitative data as means and standard deviations. Continuous variables were compared using the Mann-Whitney test. All tests were two-sided, and *p* values <0.05 were considered significant. The search for correlation was achieved through the calculation of the Spearman coefficient. The analyses were performed using the R statistical program, version 4.1.0 (R Foundation for Statistical Computing, Vienna, Austria).

## Results

### Patient’s and CPB characteristics

Twenty five consecutive patients were included during the four-month inclusion period, 13 of them were (52%) males, with a mean age of 59.2 (±15.7) years. The preoperative mean pulmonary arterial pressure (mPAP) was 39.3 (±11.5) mmHg. The mean duration of CPB, aortic cross clamp time, and DHCA were 244.9 (**±**28.8), 58.3 (**±**11.6), and 15.2 (±4.1) min, respectively. During the DHCA, the mean sweep gas inflow was 1.4 (±0.8) L/min (Table 1).

**Table 1:** Patient’s characteristics and perioperative bypass data.

Variables	Patients (*n* = 25)
Demographic characteristics	
Age (years)	59.2 (±15.7)
Male sex	13 (52)
Body Mass Index (kg/m^2^)	28.2 (±5.5)
Body surface area (m^2^)	1.9 (±0.2)
Preoperative cardiac output (L/min)	5.6 (±1.2)
Preoperative cardiac index (L/min/m^2^)	2.9 (±0.6)
Preoperative mPAP (mmHg)	39.3 (±11.5)
Preoperative capillary wedge pressure (mmHg)	10.3 (±4.5)
Preoperative TPR (dynes/sec/cm^5^)	620.3 (±262.6)
Preoperative haemoglobin level	14.1 (±2.4)
Perioperative data	
Duration of CPB (min)	244.9 (±28.8)
Aortic cross-clamp time (min)	58.3 (±11.6)
Duration of the circulatory arrest (min)	15.2 (±4.1)
Temperature during the circulatory arrest (°C)	18.4 (±0.6)
Inspired fraction of O_2_ (%)	90 (±9.6)
Sweep gas flow (L/min)	1.4 (±0.8)
Shunt line flow (L/min)	0.6 (±0.1)
Volume of the cardiotomy reservoir (mL)	2570 (±706)

Results are expressed in mean (±SD) and number (%). Volume of the cardiotomy reservoir corresponds to the volume of blood contained in the reservoir during the circulatory arrest.

### Blood gas parameters

In the alpha-stat analysis, initial pH and PaCO_2_ were 7.31 (±0.09) and 43.2 (±9.9) mmHg, respectively, and final pH and PaCO_2_ were 7.51 (±0.14); *p* < 0.001, and 23.4 (±11.9) mmHg; *p* < 0.001. In the pH-stat analysis, the mean final pH was 7.79 (**±**0.16), and the mean PaCO_2_ was 11.3 (**±**5.8) mmHg. The PaO_2_ was significantly lower at the end of the DHCA. Sodium and calcium ion concentrations were significantly lower at the end of the DHCA (139 (±) vs. 137 (±) mmol/L; *p* < 0.05 and 1.15 (±0.04) vs. 1.13 (±0.07) mmol/L; *p* = 0.03, respectively). The strong ion difference was significantly lower at the end of the DHCA (31.6 (±4.4) vs. 28.7 (±4.3) mmol/L; *p* = 0.01) (Table 2).

**Table 2:** Blood gas analysis at the beginning and the end of the circulatory arrest.

Variables	ABG at the beginning of circulatory arrest				ABG at the end of circulatory arrest		
	Alpha stat		pH stat		Alpha stat		pH stat
FiO_2_ (%)				92.7 (± 9.6)		
pH	7.31 (±0.09)		7.56 (±0.09)		7.51 (±0.14)*		7.79 (±0.16)*
PaCO_2_ (mmHg)	43.2 (±9.9)		19.5 (±4.7)		23.4 (±11.9)*		11.3 (±5.8)*
PaO_2_ (mmHg)	735 (±74)		602 (±124)		692 (±187)*		612 (±161)*
SaO_2_ (%)		99.9 (±0.16)					99.9 (±0.5)		
HCO_3_^-^ (mmol/L)		20.9 (±2.9)					19.8 (±3.9)		
Excess Base (mmol/L)		−3.5 (±5.8)					−4.5 (±3.9)		
Lactate (mmol/L)		3.3 (±1.0)					3.4 (±0.8)		
Sodium (mmol/L)		139 (±3.0)					137 (±2.9)**		
Chloride (mmol/L)		112 (±3.5)					114 (±3.5)		
Potassium (mmol/L)		4.5 (±0.8)					4.7 (±0.7)		
Calcium (mmol/L)		1.15 (±0.04)					1.13 (±0.07)**		
Strong ion difference (mmol/L)		31.6 (±4.4)					28.7 (±4.3)**		
Hemoglobin (g/dL)		9.7 (±1.0)					9.6 (±1.0)		

Results are expressed in mean (±SD). *p < 0.0001 compared to initial value. **p < 0.05 compared to initial value. PaO_2_: arterial partial pressure of O_2_; PaCO_2_: arterial partial pressure of CO_2_; SaO_2_: arterial oxygen saturation.

### Correlation analysis

**Figure 1:**
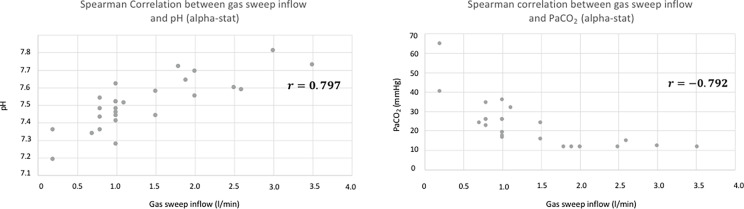
Spearman correlation analysis between pH (alpha-stat analysis), PaCO_2_ at the end of the circulatory arrest, and the gas sweep inflow set up on the CPB.

**Figure 2:**
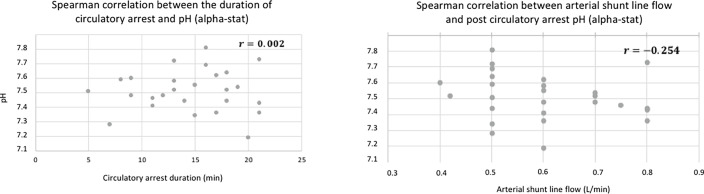
Spearman correlation analysis between first, the pH (alpha-stat analysis) at the end of the circulatory arrest and the duration of the circulatory arrest, and second, the pH (alpha-stat analysis) at the end of the circulatory arrest and the arterial shunt line flow.

There was a significant correlation between the gas sweep flow on the one hand, and the post-DHCA pH and PaCO_2_ on the other hand (*r* = 0.797; *p* < 0.0001 and *r* = −0.792; *p* < 0.0001, respectively; Figure 1). There were no correlations between the duration of DHCA and the post-DHCA pH, nor between the arterial shunt line flow and the post-DHCA pH (*r* = 0.002; *p* = 0.99 and *r* = −0.254; *p* = 0.21, respectively; Figure 2).

## Discussion

In this pilot study, we investigated the dynamic evolution of the acid-base balance and electrolyte status of the blood contained in the cardiotomy reservoir, between the beginning and the end of the deep hypothermic circulatory arrest, in patients undergoing PAE surgery.

Our results showed that the pH significantly increases and that the PaCO_2_ decreases during DHCA. The gas sweep inflow was well correlated with the post-DHCA pH, whereas the arterial shunt line flow and the duration of DHCA were not. In some cases, the pH was as high as 7.8 at the end of the DHCA (alpha-stat). The rise in pH and the drop in PaCO_2_ are related to the continuous elimination of CO_2_ from the blood contained in the cardiotomy reservoir, which circulates through the membrane oxygenator via the arterial shunt line. During DHCA, the blood contained in the reservoir is not in contact with live tissues (except blood cells), therefore its CO_2_ content will decrease as it passes through the oxygenator. Only blood cells, such as platelets and leukocytes, will produce CO_2_ through oxidative phosphorylation [10]. In two patients, when the gas sweep flow was set up below 0.5 L/min (at 0.2 L/min in both cases), the pH decreased at the end of the DHCA, suggesting that the production of CO_2_ by blood cells was higher than its removal. A sweep gas flow ranging between 0.5 and 1.0 L/min allowed for maintaining a post-DHCA PaCO_2_ above 20 mmHg and pH below 7.6 (alpha-stat), in most cases (Figure 1). The use of continuous, inline blood gas monitoring on the arterial shunt may help to monitor the variation of pH and PaCO_2_ during DHCA. The clinical implication of such high pH observed at the end of DHCA is unknown. However, brutal variations of PaCO_2_ and pH could negatively affect cerebral blood flow (CBF) and be associated with worse outcomes as has already been described following ECMO initiation [11]. The sensitivity of the cerebral vascular bed to pH-related dissolved CO_2_ variation is preserved in hypothermia, even though it seems to be decreased at 18 °C [4, 6, 7, 12, 13]. This is on the basis of the pH-stat strategy that PaCO_2_ is maintained at 40 mmHg, at temperature-corrected values, on a blood gas analyzer. pH-stat increases cerebral blood flow, the rate, and homogeneity of brain cooling during induction of hypothermia. It also decreases cerebral metabolism and allows for a better unloading of O_2_ by the hemoglobin [14]. In contrast, it could lead to an uncoupling between cerebral blood flow and metabolism, and luxury perfusion as the cerebral VO_2_ decreases by more than half of its baseline values at 18 °C [12, 15]. The perfusion of very “alkaline” blood into the patient at the end of the DHCA could potentially lead to a decrease in CBF and subsequent risk of ischemic injury. The effect of such extreme values of pH on the blood flow of other organs, such as the kidneys or liver, is poorly known. In the kidneys, some studies suggest that blood pH plays an important role in renal blood flow. Respiratory alkalosis was associated with increased vascular resistance through contraction of vascular smooth muscle and a decrease in renal blood flow [16, 17]. On the contrary, other studies found that a low blood pH and hyperchloremia were associated with decreased renal blood flow [18, 19]. Also, the Bohr effect seems to play an important role in O_2_ delivery to the kidney’s medulla, meaning that a higher pH could compromise the O_2_ supply to the kidneys [20]. Given that acute kidney injuries following PAE are frequent (up to 30–40%), limiting acid-base disorders during DHCA could have a significant impact on organ perfusion [21].

Two acid-base disturbances coexist during the period of DHCA, as a decrease of the SID was observed, in addition to the respiratory alkalosis. It could be linked to an adaptive response to counteract the rise in pH, resulting from strong ion exchange (mostly sodium) between blood cells and plasma and the accumulation of lactate. However, the change in SID in response to a change in pH is slow and limited within the red blood cells and plasma, so it cannot have a significant effect throughout the DHCA [22]. The presence of a non-respiratory acidosis has probably little effect on CBF: human and animal studies showed that changes in CSF pH in response to non-respiratory acidosis or alkalosis are very small because of effective regulatory mechanisms [23, 24]. However, there is no data on the effectiveness of these mechanisms in deep hypothermic conditions.

The main limitation of our study was that we were not able to determine the potential clinically detrimental effect linked to the reperfusion of alkaline blood after DHCA. However, the study was not designed for this purpose, the goal was to explore the change of blood pH during DHCA. Further study monitoring the blood flow of different organs following reperfusion, according to the value of blood pH contained in the cardiotomy reservoir, could be interesting. Additionally, the blood that remains in the patient’s capillaries during the DHCA becomes progressively loaded with dissolved CO_2_ and lactate because the metabolic production continues, whereas the perfusion is stopped, and this should lead to a decrease in the pH of the capillary blood. At the end of the DHCA, the alkaline blood from the reservoir and the remaining blood in the patient mix together, but the resulting pH, PaCO_2_, and effect on CBF remained unknown. We were also not able to determine the effect of the high concentration of O_2_ observed in our cohort during the reperfusion period. As far as we know, there is no specific recommendation on how to set up F_m_O_2_ during DHCA. According to our practices, the F_m_O_2_ is increased during the cooling and DHCA period to increase dissolved O_2_ and prevent potential hypoxia linked to the impaired capacity of hemoglobin to unload O_2_ in hypothermia [15, 25, 26]. Such high concentrations could increase the reperfusion injuries after DHCA. Further studies are needed to determine the optimal management of F_m_O_2_ during DHCA.

## Conclusion

A significant change in pH and PaCO_2_ of the blood contained in the cardiotomy reservoir was observed during the period of DHCA, which was correlated to the rate of sweep gas inflow. The clinical effect of such a change is not yet established. In the meantime, we suggest setting up the sweep gas inflow between 0.5 and 1.0 L/min during DHCA, to limit the variation of pH and PaCO_2_.

## Data Availability

The dataset used and/or analyzed during the current study is available from the corresponding authors on reasonable request.
